# Amygdala-cortical collaboration in reward learning and decision making

**DOI:** 10.7554/eLife.80926

**Published:** 2022-09-05

**Authors:** Kate M Wassum

**Affiliations:** 1 Department of Psychology, University of California, Los Angeles Los Angeles United States; 2 Brain Research Institute, University of California, Los Angeles Los Angeles United States; 3 Integrative Center for Learning and Memory, University of California, Los Angeles Los Angeles United States; 4 Integrative Center for Addictive Disorders, University of California, Los Angeles Los Angeles United States; Concordia University Canada; University of Oxford United Kingdom

**Keywords:** learning, memory, basolateral amygdala, orbitofrontal cortex, Pavlovian conditioning, Pavlovian-to-instrumental transfer

## Abstract

Adaptive reward-related decision making requires accurate prospective consideration of the specific outcome of each option and its current desirability. These mental simulations are informed by stored memories of the associative relationships that exist within an environment. In this review, I discuss recent investigations of the function of circuitry between the basolateral amygdala (BLA) and lateral (lOFC) and medial (mOFC) orbitofrontal cortex in the learning and use of associative reward memories. I draw conclusions from data collected using sophisticated behavioral approaches to diagnose the content of appetitive memory in combination with modern circuit dissection tools. I propose that, via their direct bidirectional connections, the BLA and OFC collaborate to help us encode detailed, outcome-specific, state-dependent reward memories and to use those memories to enable the predictions and inferences that support adaptive decision making. Whereas lOFC→BLA projections mediate the encoding of outcome-specific reward memories, mOFC→BLA projections regulate the ability to use these memories to inform reward pursuit decisions. BLA projections to lOFC and mOFC both contribute to using reward memories to guide decision making. The BLA→lOFC pathway mediates the ability to represent the identity of a specific predicted reward and the BLA→mOFC pathway facilitates understanding of the value of predicted events. Thus, I outline a neuronal circuit architecture for reward learning and decision making and provide new testable hypotheses as well as implications for both adaptive and maladaptive decision making.

## Introduction

To make good decisions we use the time machine that is our brain to cast ourselves into the future, consider the likely outcomes of our choices, and evaluate which one is currently most desirable. This time machine is programed by our memories. To know what is in the future, we often rely on the past. Previously learned associative relationships (e.g. stimulus-outcome) support decision making by enabling us to mentally simulate likely future outcomes Balleine and Dickinson, 1998a; Delamater, 2012; Fanselow and Wassum, 2015. These memories support understanding of the predictive *‘states’* that signal available or forthcoming outcomes. Such states are fundamental components of the internal model of environmental relationships, aka cognitive map Tolman, 1948, we use to generate the predictions and inferences needed for flexible, advantageous decision making Delamater, 2012; Fanselow and Wassum, 2015; Dayan and Daw, 2008; Balleine, 2019. For example, during the 2020 quarantine many of us learned that the stimuli (e.g. restaurant logos) embedded in food-delivery apps signal the availability of specific types of food (e.g. tacos, sushi, pizza). These cues allow us to mentally represent each predicted food, consider its value, and decide if it is a suitable dinner option. To ensure flexible behavior, these representations must be detailed. To choose the best dinner option, it is not sufficient to know that each leads to something ‘good’ or to ‘food’. Rather, the identifying, sensory features of each food (e.g., flavor, texture, nutritional content) must be represented. You might have just had Mexican for lunch, rending tacos undesirable. If you develop gluten intolerance, you will know to avoid pizza. After your doctor suggests increasing your Omega-3 intake, you may consider sushi a better option. Rich, *outcome-specific, appetitive, associative memories* enable expectations, ensure rapid behavioral adjustments to internal and environmental changes, and allow one to infer the most advantageous option in novel situations Balleine and Dickinson, 1998a; Delamater, 2012; Fanselow and Wassum, 2015; Delamater and Oakeshott, 2007. Failure to properly encode or use such memories can lead to absent or inaccurate reward expectations and, thus, ill-informed motivations and decisions. This is characteristic of the cognitive symptoms underlying substance use disorder and many other psychiatric conditions, including obsessive-compulsive disorder, compulsive overeating, schizophrenia, depression, anxiety, autism, and even aspects of neurodegenerative disease Hogarth et al., 2013; Morris et al., 2015; Seymour and Dolan, 2008; Alvares et al., 2014; Gleichgerrcht et al., 2010; Hogarth et al., 2013; Dayan, 2009; Voon et al., 2015; Heller et al., 2018; Chen et al., 2015; Huys et al., 2015; Culbreth et al., 2016. Thus, my broad goal here is to discuss recent findings on the neuronal systems that support outcome-specific, appetitive, associative memory and its influence on decision making.

In recent years, our understanding of the neuronal circuits of appetitive associative learning and decision making has grown dramatically. There has been considerable work on the bidirectional connections between the basolateral amygdala and orbitofrontal cortex. I review recent discoveries made about the function of this circuit using sophisticated behavioral approaches to diagnose the content of appetitive memory in combination with modern circuit dissection tools. Table 1 summarizes key findings. I focus on work in experimental rodents in which these tools have been most commonly applied, but provide some functional comparison to primates, including humans. I finish with emergent conclusions, hypotheses, and future directions.

**Table 1.** Key findings.

	Outcome-specific learning			Outcome-specific decision making			
					**Sensitivity to devaluation**		
**Hub**	**Stimulus-Outcome**	**Action-outcome**	**Incentive value**	**Pavlovian-to-instrumental transfer**	**Pavlovian responses**	**Instrumental choice**	**Incentive value**
BLA	Necessary Sias et al., 2021	Necessary Corbit et al., 2013	Necessary Malvaez et al., 2019; Parkes and Balleine, 2013; Wassum et al., 2009; Wassum et al., 2011	Necessary Sias et al., 2021; Ostlund and Balleine, 2008; Corbit and Balleine, 2005; Hatfield et al., 1996; Blundell et al., 2001; Malvaez et al., 2015, Lichtenberg et al., 2021	Necessary Sias et al., 2021; Hatfield et al., 1996; Johnson et al., 2009, Lichtenberg et al., 2021; Murray and Izquierdo, 2007; Málková et al., 1997; West et al., 2012	Necessary Parkes and Balleine, 2013; Johnson et al., 2009; Murray and Izquierdo, 2007; Balleine et al., 2003; Coutureau et al., 2009	Necessary Malvaez et al., 2019
lOFC	Necessary Sias et al., 2021	X^38^	Necessary & Sufficient Malvaez et al., 2019; Baltz et al., 2018	Necessary Ostlund and Balleine, 2007	Necessary Ostlund and Balleine, 2007	X^40^	?
mOFC	?	Necessary Bradfield et al., 2015; Bradfield et al., 2018	?	Necessary Bradfield et al., 2015; Bradfield et al., 2018	?	Necessary & Sufficient Bradfield et al., 2015; Bradfield et al., 2018; Gourley et al., 2016	Necessary & Sufficient Malvaez et al., 2019
**Pathway**							
lOFCàBLA	Necessary Sias et al., 2021	?	Necessary & Sufficient Malvaez et al., 2019	X^20^	?	?	X^22^
mOFCàBLA	?	?	X^22^	Necessary Lichtenberg et al., 2021	Necessary Lichtenberg et al., 2021	X^31^	Necessary & Sufficient Malvaez et al., 2019
BLAàlOFC	?	?	?	Necessary Sias et al., 2021	Necessary Sias et al., 2021	X^20^	?
BLAàmOFC	?	?	?	X^31^	Necessary Lichtenberg et al., 2021	X^31^	?

Pavlovian-to-instrumental transfer refers to outcome-selective Pavlovian-to-instrumental transfer; X, not necessary; ?, no evidence known to the author currently in the literature.

## Anatomy

### Basolateral amygdala

The amygdala is a highly conserved, temporal lobe, limbic system structure with basolateral, central, and medial subcomponents Duvarci and Pare, 2014; Ehrlich et al., 2009; Janak and Tye, 2015; Sah et al., 2003; LeDoux, 2007. I focus on the basolateral amygdala (BLA) which consists of lateral, basal, and basomedial nuclei and contains glutamatergic principle neurons, inhibitory interneurons, and potentially GABAergic projection neurons Birnie et al., 2022. GABAergic intercalated cells flank the BLA Ehrlich et al., 2009; Marowsky et al., 2005. The BLA is heavily innervated by glutamatergic projections from sensory thalamus and cortex McDonald and Jackson, 1987; Ledoux et al., 1987; Linke et al., 2000; McDonald, 1998. It also receives midbrain monoaminergic input Sadikot and Parent, 1990; Lutas et al., 2019; Brinley-Reed and McDonald, 1999; Fallon and Ciofi, 1992. The BLA sends unidirectional projections to the central amygdala, ventral and dorsal striatum, and the bed nucleus of the stria terminalis Kelley et al., 1982; Kita and Kitai, 1990; McDonald, 1991b; McDonald, 1991a. The glutamatergic projections between the BLA and cortex are reciprocal, positioning the BLA to both influence and be influenced by cortical activity. Thus, the BLA is a site of anatomical convergence well positioned to influence the activity of the broader learning and decision-making circuit.

### Orbitofrontal cortex

The orbitofrontal cortex (OFC) is a prefrontal cortical region in the ventral frontal lobe Izquierdo, 2017; Hoover and Vertes, 2011; Heilbronner et al., 2016. OFC structure differs between rodents and primates, in particular, granular cortex (dense granular cells in layer IV), which rodents lack Preuss, 1995. But rodent OFC has anatomical and functional homology with portions of primate OFC (Heilbronner et al., 2016; Price, 2007; Rudebeck and Izquierdo, 2022). The OFC is divided into lateral (lOFC) and medial (mOFC) subdivisions. The lOFC, as opposed to mOFC, receives inputs from sensory-processing regions Carmichael and Price, 1995; Ongür and Price, 2000. There is also evidence of distinct connectivity based on the anterior-posterior axis Barreiros et al., 2021. The OFC has many cortico-cortico connections Carmichael and Price, 1995; Ongür and Price, 2000. It also receives input from the hippocampus and midbrain Ongür and Price, 2000; Barreiros et al., 2021. The OFC is reciprocally connected with mediodorsal thalamus, hypothalamus, and amygdala Lichtenberg et al., 2021; Ongür and Price, 2000; Barreiros et al., 2021. Among the OFC outputs are critical projections to the striatum, with anatomical segregation between OFC subregions Heilbronner et al., 2016. Thus, lOFC and mOFC are well positioned to detect associations between external and internal information and to support learning and decision making within a broad network.

### Orbitofrontal cortex-basolateral amygdala circuit

Owing to their well-documented, dense, excitatory, bidirectional connections reported in both rodents and primates Malvaez et al., 2019; Lichtenberg et al., 2021; Kita and Kitai, 1990; Hoover and Vertes, 2011; Heilbronner et al., 2016; Barreiros et al., 2021; Reppucci and Petrovich, 2016; Lichtenberg et al., 2017; Morecraft et al., 1992, the BLA and OFC are long-standing collaborators. Both lOFC and mOFC send dense intermingled projections across the anterior-posterior extent of the BLA Malvaez et al., 2019. The BLA also projects back to both lOFC and mOFC, with lOFC-projectors being slightly more prominent in anterior BLA Lichtenberg et al., 2021. The BLA pathways to mOFC and lOFC are largely distinct, with very few BLA neurons collateralizing to both lOFC and mOFC Lichtenberg et al., 2021. Thus, the BLA and OFC are well positioned to engage in bidirectional communication.

## Basolateral amygdala function

The BLA is widely known as a processing hub for emotionally significant events. Such events are major contributors to learning and decision making and, thus, the BLA is a good entry point to understanding the neuronal circuits of such processes. The BLA’s function in aversive emotional learning has been well demonstrated. BLA lesion or inactivation severely disrupts the acquisition and expression of conditional fear and active avoidance Davis and Smith, 1992; Fanselow and LeDoux, 1999; Killcross et al., 1997; Lázaro-Muñoz et al., 2010. By contrast, such manipulations have little or no effect on general measures of appetitive Pavlovian (e.g. goal- or cue approach responses to reward-predictive stimuli) or instrumental (e.g. pressing a lever that earns reward) behavior Wassum and Izquierdo, 2015. This has led to the notion that the BLA is a brain locus for fear.

But the BLA does way more than fear. Null effects of BLA manipulations can arise because behavior can be guided by multiple different control systems. Humans and other animals can encode the relationship between a Pavlovian cue and the specific outcome it predicts (*stimulus-outcome*), as well as an instrumental action and the outcome it earns (*action-outcome*). These associative memories contribute to an internal model of the structure of an environment that enables predictions and inferences for flexible, advantageous decision making Delamater, 2012; Fanselow and Wassum, 2015; Dayan and Daw, 2008; Balleine, 2019; Doll et al., 2012, for example, considering which dinner option to choose based on current circumstances. However, this is not the only type of memory we form. For example, we and other animals also form habits Balleine, 2019; Sutton and Barto, 2022; Malvaez and Wassum, 2018, response policies performed relatively automatically based on their past success without forethought of their consequences, e.g., always order pizza on Fridays. Specific predicted outcomes are not encoded in this memory system Balleine, 2019; Sutton and Barto, 2022; Malvaez and Wassum, 2018. General Pavlovian or instrumental behaviors do not typically require any consideration of their specific outcome, so they can be controlled by either system. Thus, BLA lesion or inactivation could shift behavioral control strategy without any ostensible effect on behavior.

Using tests that reveal the content of associative memory and, thus, behavioral control system guiding behavior, the BLA has been shown to play a fundamental role in encoding, updating, and retrieving detailed, outcome-specific reward memories critical for the predictions and inferences that support flexible decision making Wassum and Izquierdo, 2015; Chesworth and Corbit, 2017; Balleine and Killcross, 2006. The most canonical of these tests is *outcome-selective devaluation*. When making a decision, we consider the current value of the potential outcome. If using a stimulus-outcome or action-outcome memory, we will reduce performance of a behavior when its outcome has been devalued by selective satiation or pairing with illness. This will occur even without the opportunity to learn that the particular behavior leads to a devalued outcome. Memories of the predicted reward allow inferences about how advantageous it would be to pursue. For example, you can infer Mexican might not be great for dinner if you just had tacos for lunch (sensory-specific satiety) or you will avoid ordering sushi from a particular restaurant if you became ill the last time you had it (conditioned taste aversion). Similarly, animals will press less on a lever that earns a devalued outcome relative to a valuable reward, or will show fewer food-port approach responses to a cue signaling a devalued outcome relative to a valuable one. Although BLA lesion or inactivation does not disrupt general Pavlovian or instrumental behavior, it does render these behaviors insensitive to post-training devaluation of the predicted outcome Parkes and Balleine, 2013; Hatfield et al., 1996; Johnson et al., 2009; Murray and Izquierdo, 2007; Málková et al., 1997; West et al., 2012; Balleine et al., 2003; Coutureau et al., 2009; Pickens et al., 2003. Thus, the BLA is important for stimulus-outcome and action-outcome memory.

The BLA also helps to learn the value of a reward and adapt decisions accordingly. A reward’s value as an incentive is dependent on current motivational state. For example, a food item has a high value and incentivizes robust pursuit when hungry, but low value supporting less pursuit when sated. This incentive information is encoded during experience in a relevant motivational state (i.e. *incentive learning;* Wassum et al., 2011; Dickinson and Balleine, 1994; Dickinson and Balleine, 1990; Balleine et al., 1995). For example, if a friend serves you pizza when you are hungry, you will learn that pizza is delicious and satisfying (i.e. valuable) when you are hungry and will be more likely to order it yourself when hungry again in the future. Likewise, after being trained sated to lever press for a particular food reward, non-continent experience with that food while hungry will cause animals to increase pressing when they are hungry subsequently. The converse is also true; after experiencing a particular food when sated, animals will decrease actions that earn that food when they are sated again in the future. The BLA mediates such incentive learning Malvaez et al., 2019; Parkes and Balleine, 2013; Wassum et al., 2009; Wassum et al., 2011.

In both these cases, the value manipulation is outcome specific. For example, having tacos for lunch will make you less inclined to select them for dinner, but will not affect the desirability of pizza or sushi. What you learn about the pizza at your friend’s house is unlikely to change your decisions for sushi or tacos. Likewise, changes to the value of one food reward (e.g. sucrose) by feeding to satiety, pairing with malaise, or experiencing it while hungry, will primarily affect behaviors for that specific and not other foods (e.g. pellets; Dickinson and Balleine, 1994). Thus, the BLA is critical for detailed, outcome-specific reward memory.

Further supporting BLA function in outcome-specific reward memory is evidence that the BLA is required for *outcome-specific Pavlovian-to-instrumental transfer* (PIT) Ostlund and Balleine, 2008; Corbit and Balleine, 2005; Hatfield et al., 1996; Blundell et al., 2001; Malvaez et al., 2015. Subjects first learn that two different cues each predict a unique food reward (e.g., pellets or sucrose) and, separately, that they can press on one lever to earn one of the foods and another lever to earn the other. The PIT test assesses the ability to use the cues to mentally represent which specific reward is predicted and use this to motivate choice of the action known to earn that same unique reward Kruse et al., 1983; Colwill and Motzkin, 1994; Gilroy et al., 2014; Corbit and Balleine, 2016. This is consistent with the notion that the subjects use the cue to infer which reward is more likely to be available and, thus, which action is most advantageous. For example, a billboard advertising an appetizing pizza on your way home may make you think about pizza and order it for dinner instead of tacos or sushi. Pre- or post-training BLA lesions will disrupt the expression of outcome-specific PIT Ostlund and Balleine, 2008; Corbit and Balleine, 2005; Hatfield et al., 1996; Blundell et al., 2001; Malvaez et al., 2015. BLA lesion will not, however, prevent cues from motivating behavior more broadly. For example, the BLA is not needed for general Pavlovian-to-instrumental transfer in which, absent the opportunity to seek out the specific predicted reward, a cue will invigorate performance of an action that earns a different reward (although typically one of the same class, e.g. food) Corbit and Balleine, 2005. Thus, the BLA is critical when adaptive appetitive behavior requires a detailed representation of a specific predicted outcome Janak and Tye, 2015; Wassum and Izquierdo, 2015; Balleine and Killcross, 2006.

Recent evidence indicates that the BLA contributes to both forming and using outcome-specific reward memories. During appetitive Pavlovian conditioning, BLA principle neurons are robustly activated at the time of stimulus-outcome pairing (reward delivery during the cue) Sias et al., 2021; Crouse et al., 2020. This activity is necessary for outcome-specific, appetitive associative memories to be formed, so that they can later influence decision making Sias et al., 2021. Similarly, BLA glutamate activity tracks the encoding of a reward’s value Malvaez et al., 2019. BLA NMDA Malvaez et al., 2019; Parkes and Balleine, 2013 and mu opioid receptors Wassum et al., 2009; Wassum et al., 2011 support such incentive learning. Thus, the BLA is activated by rewarding events and this is necessary to link the specific reward to the associated cue and to encode its incentive value. Following conditioning, the BLA is activated by reward-predictive cues Sias et al., 2021; Malvaez et al., 2015; Lutas et al., 2019; Crouse et al., 2020; Schoenbaum et al., 1998; Tye and Janak, 2007; Paton et al., 2006; Belova et al., 2008; Sugase-Miyamoto and Richmond, 2005; Beyeler et al., 2016; Schoenbaum et al., 1999; Muramoto et al., 1993; Tye et al., 2008; Beyeler et al., 2018. During the cue, transient outcome-specific BLA glutamate signals selectively precede and predict choice of the action that earns the predicted reward Malvaez et al., 2015. Correspondingly, the BLA is required to use outcome-specific stimulus-outcome memories to guide adaptive behavior and choice (e.g. express PIT) Ostlund and Balleine, 2008; Malvaez et al., 2015; Johnson et al., 2009; Lichtenberg and Wassum, 2017. BLA glutamate activity prior to bouts of reward seeking Wassum et al., 2012 also reflects the learned value of the predicted reward Malvaez et al., 2019; Wassum et al., 2012 and activation of both BLA NMDA and AMPA receptors is necessary for value-guided reward-seeking Malvaez et al., 2019. Thus, the BLA is activated by cues and during decision making and this activity is critical for using information about the predicted reward to guide choice.

These data indicate that the BLA mediates both the formation of outcome-specific reward memories and the use of these memories to inform decision making. They also suggest the BLA is important for using states to predict information about associated rewards. Stimulus-outcome memories are state-dependent: the external cue sets the state predicting a specific rewarding outcome. Incentive value is gated by motivational state. Internal physiological conditions dictate the incentive value of a particular reward. Thus, BLA activity is critical for linking specific rewarding events to the states, defined by both by external cues and internal physiological signals, with which they are associated and for using those memories to guide adaptive reward pursuit choices.

Other recent evidence also supports a role for the BLA in appetitive learning and decision making. For example, optical inhibition of BLA neurons disrupts risky decision making Orsini et al., 2017. When applied prior to choice, BLA inhibition will decrease choices of the larger risky reward Orsini et al., 2017, likely by preventing the subject from retrieving the incentive value of that large reward. This can also occur with less temporally-specific BLA inactivation Ghods-Sharifi et al., 2009. When applied during outcome experience, BLA inhibition will promote risky decision making, perhaps by preventing encoding of the punishing outcome Orsini et al., 2017 or by forcing learning to occur via another, less punishment-sensitive system. Indeed, post-training BLA lesions will also increase risky choice Zeeb and Winstanley, 2011; Orsini et al., 2015 and chemogenetic BLA inhibition prevents learning from positive or negative outcomes to update cue-response strategies Stolyarova et al., 2019.

The BLA also encodes information relevant for learning and using state-dependent, outcome-specific reward memories. BLA neurons can signal the unsigned Roesch et al., 2010; Esber et al., 2012, positive, or negative Esber and Holland, 2014 prediction errors that support learning. Populations of BLA neurons can reflect taste-specific gustatory information Fontanini et al., 2009 and respond selectively to unique food rewards Liu et al., 2018; Courtin et al., 2022, which could support the generation of outcome-specific reward memories. In both rodents and primates, BLA neuronal responses to predictive cues can encode the value of the predicted reward Schoenbaum et al., 1998; Paton et al., 2006; Belova et al., 2008; Jenison et al., 2022; Saddoris et al., 2005; Belova et al., 2007, inferences about reward magnitude Lucantonio et al., 2015, prospectively reflect goal plans Hernádi et al., 2015, and predict behavioral choices Grabenhorst et al., 2012. BLA neurons also encode state-dependent exploratory behaviors in distinct neuronal ensembles Fustiñana et al., 2021. Thus, during decision making BLA activity reflects critical state-dependent decision variables. The extent to which BLA neuronal ensembles encode outcome-specific predictions during decision making is an exciting open question.

Both reward learning and expectation signals have also been detected in human amygdala Elliott et al., 2004; Hampton et al., 2007; Yacubian et al., 2006, with some evidence that these occur in BLA in particular Prévost et al., 2011. BLA activity in humans also relates to the ability to use an internal model of environmental structure to guide decision making Prévost et al., 2013, including the ability to use cues to generate the outcome-specific reward expectations that influence PIT Prévost et al., 2012. Thus, BLA function in learning and using outcome-specific reward memories is conserved in humans.

### Orbitofrontal cortex → basolateral amygdala pathway

The OFC is a likely candidate for supporting the BLA’s function in forming state-dependent, outcome-specific reward memories and using them to guide decision making. It has been implicated in both learning and using information about rewarding events to inform flexible decision making Wilson et al., 2014; Schuck et al., 2016; Bradfield and Hart, 2020; Shields and Gremel, 2020; Sharpe et al., 2019; Wikenheiser and Schoenbaum, 2016; Rudebeck and Rich, 2018; Gardner and Schoenbaum, 2020. Like the BLA, OFC lesion or inactivation does not disrupt general Pavlovian conditional approach responses but does render this behavior insensitive to devaluation of the predicted outcome Ostlund and Balleine, 2007; Pickens et al., 2003; Gallagher et al., 1999; Pickens et al., 2005. The OFC is also required to use cues to both bias choice in the PIT test Ostlund and Balleine, 2007 and to make inferences about available reward Jones et al., 2012. Thus, much like the BLA, the OFC is critical for using cues to represent future possible rewards and inform predictions and inferences about how advantageous a particular course of action might be. Such findings have contributed to the notion that the OFC is a critical element in the brain’s cognitive map Wilson et al., 2014; Schuck et al., 2016; Bradfield and Hart, 2020; Shields and Gremel, 2020; Sharpe et al., 2019; Wikenheiser and Schoenbaum, 2016; Rudebeck and Rich, 2018, an internal model of the associative relationships (e.g. stimulus-outcome) within an environment required for mentally simulating future potential outcomes to inform decisions. The OFC may achieve this function via its interactions with the BLA. Indeed, as described above, the BLA also mediates the formation and use of the state-dependent reward memories that contribute to cognitive maps. Both lOFC and mOFC participate in appetitive behavior, though have unique functions Izquierdo, 2017; Bradfield and Hart, 2020; Wallis, 2011. Accordingly, recent evidence indicates unique functions of lOFC→BLA and mOFC→BLA projections.

### Lateral orbitofrontal cortex → basolateral amygdala pathway

The lOFC→BLA pathway helps to link specific rewarding events to predictive states. Optical inhibition of lOFC→BLA projections during stimulus-outcome pairing attenuates the encoding of specific stimulus-outcome memories as evidenced by the inability of subjects to later use those memories to allow cues to bias choice behavior during a PIT test Sias et al., 2021. Similarly, inhibition of lOFC→BLA projections attenuates encoding of the positive incentive value of a particular food reward Malvaez et al., 2019. Thus, lOFC→BLA pathway activity mediates encoding of state-dependent, outcome-specific reward memories. lOFC→BLA activity is also sufficient to drive subjects to assign a high value to a particular reward Malvaez et al., 2019. Pairing optical stimulation of lOFC→BLA projections with non-contingent experience of a food reward causes animals to subsequently seek out that specific food, but not other foods, more vigorously. Thus, lOFC→BLA pathway activity is capable, at least in part, of elevating the incentive value of a specific reward, information that later informs reward-seeking decisions. Together these data indicate that lOFC via its direct projections to the BLA mediates the ability to link rewarding events to the external and internal states with which they are associated and, thus, regulates the formation of an internal model, aka cognitive map, that enables the predictions and inferences needed for flexible, advantageous decision making.

This is consistent with evidence that lOFC is important for learning about rewarding events. The lOFC mediates incentive learning Baltz et al., 2018 and helps link cues to their value in dynamic learning environments Noonan et al., 2010; Walton et al., 2010; Chau et al., 2015; Noonan et al., 2017. It is also consistent with evidence, across species, that lOFC can encode high-dimensional, outcome-specific representations of predicted rewards and their value Wilson et al., 2014; Rudebeck and Rich, 2018; McDannald et al., 2014; Howard et al., 2015; Klein-Flügge et al., 2013; Gottfried et al., 2003; Howard and Kahnt, 2017; Rich and Wallis, 2016; Farovik et al., 2015; Lopatina et al., 2015; Suzuki et al., 2017; Rudebeck and Murray, 2014. lOFC neurons respond to rewarding events during learning to signal reward expectations that may support learning in downstream structures, such as the BLA Stalnaker et al., 2018b; Stalnaker et al., 2018a. Indeed, OFC lesion disrupts expected outcome and decision-related activity in BLA Wassum et al., 2012; Saddoris et al., 2005; Lucantonio et al., 2015.

lOFC→BLA projections do not mediate the retrieval of reward memories or use of this information to guide decisions. Chemogenetic inhibition of lOFC→BLA projections does not disrupt value-guided reward seeking Malvaez et al., 2019 or the ability to use reward cues to bias choice (express PIT) Lichtenberg et al., 2017. Stimulation of this pathway will not promote reward seeking Malvaez et al., 2019. Thus, lOFC→BLA projections mediate the encoding, but not retrieval or use of state-dependent reward memories. This is not to imply that the lOFC does not participate in using reward memories to guide decision making. It does Ostlund and Balleine, 2007; Pickens et al., 2005; Jones et al., 2012; Howard et al., 2020; West et al., 2018. This function is likely to be achieved via projections other than those to the BLA, for example to the striatum Hoover and Vertes, 2011; Gremel and Costa, 2018; Gremel et al., 2016; Gourley et al., 2013.

This conclusion seemingly contradicts evidence that optical inhibition of lOFC→BLA projections disrupts cue-induced reinstatement of cocaine seeking Arguello et al., 2017, ostensibly a task in which cue-drug memory influences drug seeking. This effect could be due to unintended inhibition of collateral projections to other brain regions. However, it is more easily reconciled by considering that cue-induced reinstatement contains a learning process: action reinforcement by drug cues. This conditional reinforcement could be mediated by lOFC→BLA projections.

The lOFC→BLA pathway also supports performance in more dynamic learning and decision scenarios. For example, lOFC→BLA lesion influences performance during reversal learning, in which subjects must learn, integrate, and use information about reward availability and option value Groman et al., 2019a. The above evidence from tasks that parse learning and retrieval processes suggests that lOFC→BLA projections may primarily support reward learning in such dynamic scenarios.

### Medial orbitofrontal cortex → basolateral amygdala pathway

In contrast to the lOFC→BLA pathway, mOFC→BLA projections do regulate the influence of reward memories over decision making. mOFC→BLA projection activity is critical for using environmental cues to know which specific reward is predicted and the current value of that option. Chemogenetic inactivation of this pathway disrupts the ability to use reward cues to guide choice during an outcome-specific PIT test and prevents subjects from adapting cue responses following selective devaluation of the predicted reward Lichtenberg et al., 2021. mOFC→BLA projections are also necessary for using the previously encoded incentive value of an expected reward to ensure its adaptive pursuit Malvaez et al., 2019. Stimulation of this pathway can even facilitate the ability to use a subthreshold reward value memory to incentivize seeking of a specific reward Malvaez et al., 2019. Thus, mOFC→BLA projections mediate the use of the current state, defined both by external cues and internal physiological signals, to inform decision making. In each above experiment, the tests were non-reinforced, forcing subjects to use their memories of the predicted rewards to guide decisions. When such memories are not required or have not been encoded, mOFC→BLA projection activity is dispensable Malvaez et al., 2019. mOFC→BLA projections, therefore, mediate the use of state-dependent, outcome-specific reward memories to guide decisions.

This is consistent with evidence that mOFC itself participates in appetitive decision making Malvaez et al., 2019; Bradfield et al., 2015; Bradfield et al., 2018; Gourley et al., 2016; Noonan et al., 2010; Noonan et al., 2017; Stopper et al., 2014; Münster and Hauber, 2018; Dalton et al., 2016; Bray et al., 2010; Rudebeck and Murray, 2011; Yamada et al., 2018 and is especially important for using knowledge of the structure of the environment to make predictions about currently unobservable events Bradfield et al., 2015. It also accords with data that mOFC represents general information about expected events that is used to make decisions based on value estimations or comparisons Suzuki et al., 2017; Rudebeck and Murray, 2011; Lopatina et al., 2016; Burton et al., 2014; Kennerley et al., 2011; Plassmann et al., 2010; Levy and Glimcher, 2011; Lopatina et al., 2017; Padoa-Schioppa and Assad, 2006; Pritchard et al., 2005. These data suggest that mOFC’s function in representing future events to guide decision making is, at least in part, achieved via direct projections to BLA.

Although critical for using state-dependent reward memories to guide decision making, the mOFC→BLA pathway is not needed to encode these memories. Chemogenetic inactivation of mOFC→BLA projections does not disrupt incentive learning, and optical activation of this pathway will not promote value encoding Malvaez et al., 2019. Thus, lOFC→BLA and mOFC→BLA pathway function in forming and using reward memories is doubly dissociable. This specialization of OFC→BLA pathways for learning associative information (lOFC→BLA) v. using it to make decisions (mOFC→BLA) is consistent with similar evidence of lOFC v. mOFC encoding v. decision functions in non-human primates and humans Noonan et al., 2010; Noonan et al., 2017. The primate lOFC has been shown to be involved in credit assignment Noonan et al., 2017; Rudebeck and Murray, 2011 and value updating following devaluation Murray et al., 2015. Whereas primate mOFC has been implicated in value-guided decision making Noonan et al., 2017; Rudebeck and Murray, 2011. These functions are achieved, at least in part, via projections to the BLA. Together these data indicate that the lOFC→BLA pathway mediates the formation of state-dependent, outcome-specific reward memories and the mOFC→BLA pathway facilitates the use of this information to guide adaptive reward-related decisions.

## Basolateral amygdala → orbitofrontal cortex pathway

Projections back to the OFC are likely candidates for the BLA output pathways responsible for using state-dependent, outcome-specific appetitive memories to guide decision making. Indeed, the OFC-BLA circuit is bidirectional and the OFC has been implicated using knowledge of the associative relationships within an environment to inform the predictions and inferences necessary for flexible decision making Wilson et al., 2014; Schuck et al., 2016; Bradfield and Hart, 2020; Shields and Gremel, 2020; Sharpe et al., 2019; Wikenheiser and Schoenbaum, 2016; Rudebeck and Rich, 2018; Gardner and Schoenbaum, 2020. Pathway-specific BLA→OFC manipulations indicate these functions are facilitated, in part, via input from the BLA and are distinct between the BLA→lOFC and BLA→mOFC pathways.

### Basolateral amygdala → lateral orbitofrontal cortex pathway

BLA→lOFC projections mediate the ability to use state-dependent, outcome-specific stimulus-outcome memories to guide reward-seeking decisions. Chemogenetic inactivation of this pathway disrupts the ability to use reward cues to guide choice behavior during a PIT test and to adapt cue responses following devaluation of a predicted reward Lichtenberg et al., 2017. lOFC→BLA projections are particularly important when predicted outcomes are not readily observable and memories of environmental relationships must be used to guide decisions Lichtenberg et al., 2017. Thus, BLA→lOFC projections are critical for using stimulus-outcome memories to inform decision making, including the identity and current desirability of the predicted reward. Whether BLA→lOFC function in value is secondary to representing reward identity (if you do not know which reward is predicted, then you cannot represent its value) is a critical open question.

BLA→lOFC projection function in using stimulus-outcome memories to enable cues to inform decision making is consistent with evidence that the BLA itself is activated by reward-predictive cues Sias et al., 2021; Malvaez et al., 2015; Lutas et al., 2019; Crouse et al., 2020; Schoenbaum et al., 1998; Tye and Janak, 2007; Paton et al., 2006; Belova et al., 2008; Sugase-Miyamoto and Richmond, 2005; Beyeler et al., 2016; Schoenbaum et al., 1999; Muramoto et al., 1993; Tye et al., 2008; Beyeler et al., 2018 and necessary for using outcome-specific, stimulus-outcome memories to guide adaptive behavior and choice Ostlund and Balleine, 2008; Malvaez et al., 2015; Johnson et al., 2009; Lichtenberg and Wassum, 2017. This BLA function is mediated, at least in part, via BLA→lOFC projections. lOFC is critical for using stimulus-outcome memories to inform flexible reward-related behaviors and choice Ostlund and Balleine, 2007; Pickens et al., 2003; Gallagher et al., 1999; Pickens et al., 2005 and can encode high-dimensional rewarding representations Wilson et al., 2014; Rudebeck and Rich, 2018; McDannald et al., 2014; Howard et al., 2015; Klein-Flügge et al., 2013; Gottfried et al., 2003; Howard and Kahnt, 2017; Rich and Wallis, 2016; Farovik et al., 2015; Lopatina et al., 2015; Suzuki et al., 2017; Rudebeck and Murray, 2014. This is likely achieved via direct input from the BLA. Indeed, BLA lesion will disrupt outcome encoding in lOFC Schoenbaum et al., 2003b.

The lOFC and BLA are well positioned to collaborate in a bidirectional circuit to form (lOFC→BLA) and subsequently use (BLA→lOFC) outcome-specific reward memories. This was recently tested using a pathway-specific, serial, circuit disconnection, achieved by multiplexing unilateral optogenetic inhibition of lOFC→BLA projections during stimulus-outcome learning with unilateral, contralateral chemogenetic inhibition of BLA→lOFC projections during the use of those memories at a PIT test. This indicated that the outcome-specific associative information that requires lOFC→BLA projections to be encoded also requires activation of BLA→lOFC projections to be used for decision making. Thus, lOFC→BLA→lOFC is a functional learning and decision circuit. lOFC→BLA projections regulate the encoding of state-dependent, outcome-specific reward memories and BLA→lOFC projections mediate the subsequent use of these memories for adaptive decision making.

### Basolateral amygdala → medial orbitofrontal cortex pathway

The BLA→mOFC pathway also mediates BLA function in using reward memories to influence decisions, but differently than the BLA→lOFC pathway. Unlike BLA→lOFC, chemogenetic inactivation of BLA→mOFC projections does not disrupt the expression of outcome-specific PIT Lichtenberg et al., 2021. The BLA→mOFC pathway is, therefore, not required to retrieve outcome-specific stimulus-outcome memories or use them to influence decision making. BLA→mOFC inactivation does, however, prevent subjects from adapting cue responses following devaluation of the predicted reward Lichtenberg et al., 2021. Thus, the BLA→mOFC pathway is critical for using cues to represent the value, but not identity, of future rewards. This value information is critical for inferring how advantageous it would be to respond to the cue.

BLA→mOFC pathway function in adapting behavior based on the current value of a predicted reward is consistent with evidence that the BLA itself is needed for the sensitivity of cue responses to devaluation Ostlund and Balleine, 2008; Johnson et al., 2009 and with evidence that BLA neuronal responses to cues can represent the value of the predicted reward Schoenbaum et al., 1998; Paton et al., 2006; Belova et al., 2008; Saddoris et al., 2005; Belova et al., 2007. This function is achieved, at least in part, via BLA→mOFC projections. mOFC is itself critical, across species, for appetitive decision making Malvaez et al., 2019; Bradfield et al., 2015; Bradfield et al., 2018; Noonan et al., 2010; Noonan et al., 2017; Stopper et al., 2014; Münster and Hauber, 2018; Dalton et al., 2016, especially when the value of rewarding options must be mentally simulated Bradfield et al., 2015; Bray et al., 2010 and/or compared Gourley et al., 2016; Noonan et al., 2010; Stopper et al., 2014; Rudebeck and Murray, 2011; Yamada et al., 2018. mOFC neuronal activity can represent a cue-reward memory Namboodiri et al., 2019 and unobservable future states Lopatina et al., 2017; Elliott Wimmer and Büchel, 2019. The mOFC can also represent general information about expected events to make value estimations Suzuki et al., 2017; Rudebeck and Murray, 2011; Lopatina et al., 2016; Burton et al., 2014; Kennerley et al., 2011; Plassmann et al., 2010; Levy and Glimcher, 2011; Lopatina et al., 2017; Padoa-Schioppa and Assad, 2006; Pritchard et al., 2005. BLA→mOFC projections might facilitate the ability to use cues to generate value estimations in mOFC, at least for deciding whether or not to respond to a cue.

The function of the BLA→mOFC pathway is different from the mOFC→BLA pathway. mOFC→BLA projections are critical for using predictive states to know which specific reward is predicted and the current value of that option Malvaez et al., 2019; Lichtenberg et al., 2021. BLA→mOFC projections are only needed for the latter Lichtenberg et al., 2021. Whether BLA and mOFC function in a bidirectional circuit, like the lOFC-BLA circuit, is an important open question. For example, do mOFC→BLA projections enable BLA→mOFC projection function in using cues to adapt behavior based on the value of the predicted reward, or vice versa? This is plausible, if not likely, given that both mOFC→BLA and BLA→mOFC projections are needed for this behavior. But the BLA→mOFC pathway is unlikely to contribute to mOFC→BLA function in using cues to predict reward identity. This mOFC→BLA function is likely achieved via another BLA output, perhaps that to lOFC which is also needed for such predictions Lichtenberg et al., 2017. Another important open question is whether the BLA→mOFC pathway mediates the use of internal state-dependent incentive value, like the mOFC→BLA pathway. BLA→mOFC projections have thus far only been studied in the context of external states.

Together these data indicate that BLA outputs to the OFC mediate the ability to use stimulus-outcome memories to influence adaptive reward choices. The BLA→lOFC pathway allows one to use cues to predict specific available rewards, whereas BLA→mOFC pathway enables predictions of the value of forthcoming events. The extent to which BLA→lOFC and BLA→mOFC pathways participate in encoding reward memories is a ripe question for future investigation.

## What the orbitofrontal cortex – basolateral amygdala circuit does *not* do

Although the boundary conditions of OFC-BLA function remain to be fully delineated, emerging evidence suggests the OFC-BLA circuit may specialize in learning about and using states to make predictions about available rewards and their value, information that supports flexible decision making.

The OFC-BLA circuit is not necessary for the acquisition or expression of general conditional response policies. Inactivation of neither OFC→BLA, nor BLA→OFC pathways prevents subjects from approaching the goal location (e.g. food-delivery port) during a cue Lichtenberg et al., 2021; Lichtenberg et al., 2017. This is consistent with evidence that neither the BLA, lOFC, nor mOFC is needed for this behavior Corbit and Balleine, 2005; Hatfield et al., 1996; Malvaez et al., 2015; Bradfield et al., 2015; Bradfield et al., 2018; Everitt et al., 2000; Parkinson et al., 2000; Morse et al., 2020. Although influenced by positive outcome valence, such general cue responses do not require an outcome expectation and can be executed via a previously learned response policy that relies instead on past success. The BLA-OFC circuit is not necessary for stamping in or expressing such a response policy and, therefore, is not simply necessary for assigning valence to predictive events. Rather the BLA-OFC circuit is critical when one must use cues to access a representation of the predicted reward to support reward pursuit or decision making.

Thus far, the OFC-BLA circuit has not been found to be important for accessing knowledge of the specific consequences of an instrumental action (i.e. action-outcome memories). OFC-BLA pathway manipulations do not to affect general instrumental activity, consistent with evidence from BLA and OFC lesions Ostlund and Balleine, 2008; Corbit and Balleine, 2005; Murray and Izquierdo, 2007; Balleine et al., 2003; Ostlund and Balleine, 2007 and BLA-OFC disconnection Fiuzat et al., 2017; Baxter et al., 2000; Zeeb and Winstanley, 2013. BLA→lOFC, BLA→mOFC, or mOFC→BLA pathway inactivation also does not disrupt sensitivity of instrumental choice to devaluation of one of the predicted rewards Lichtenberg et al., 2021; Lichtenberg et al., 2017. Thus, these pathways are not needed to retrieve or use simple action-outcome memories. Both BLA and mOFC are required for this Ostlund and Balleine, 2008; Johnson et al., 2009; Balleine et al., 2003; Bradfield et al., 2015; Bradfield et al., 2018; Gourley et al., 2016. They likely achieve this function via alternate projections, perhaps those to the striatum Corbit et al., 2013; Gremel and Costa, 2018; Gremel et al., 2016; Morse et al., 2020; van Holstein et al., 2020, a region heavily implicated in action-outcome memory Malvaez and Wassum, 2018; Malvaez et al., 2018; Malvaez, 2020; Yin et al., 2005. It remains unknown whether lOFC→BLA projections are important for sensitivity of instrumental choice to devaluation. This is unlikely because lOFC→BLA projections are not needed for other tasks that require action-outcome and outcome value information Malvaez et al., 2019; Lichtenberg et al., 2017 and this pathway has generally been found to be primarily important for learning, rather than using, reward memories. The lOFC is also itself not required for sensitivity of instrumental choice to devaluation Parkes et al., 2018; Ostlund and Balleine, 2007. The lOFC is, however, involved in action-outcome memory. It becomes needed for sensitivity of instrumental choice to devaluation after action-outcome contingencies have been switched Parkes et al., 2018. This nuanced function in action-outcome memory may rely on lOFC function in state-dependent memory. After the contingencies change, one must use the latent state to know which set of action-outcome contingencies are at play. This may also explain why lOFC-BLA disconnection will disrupt choice behavior following a degradation of one action-outcome contingency Zimmermann et al., 2017. Thus, a critical open question is whether components of the OFC-BLA circuit contribute to action-outcome memory by facilitating the use of states to retrieve current action-outcome relationships.

That OFC-BLA circuitry is not necessary for the sensitivity of instrumental choice to outcome devaluation (at least in its simple form) ostensibly contradicts evidence from BLA-OFC disconnections Fiuzat et al., 2017; Baxter et al., 2000; Zeeb and Winstanley, 2013. Using cross lesions to disconnect OFC and BLA, these studies demonstrate OFC-BLA connectivity is critical for adapting choices following post-training devaluation of the predicted reward. There are three ways to reconcile these findings. First, cross lesions will disconnect both direct and multisynaptic OFC-BLA connections. The broader effects of OFC-BLA disconnection could be via the multisynaptic connections. Second, cross lesions disrupt the devaluation learning process, which is spared with more temporally-restricted manipulations. This may account for their effects on later choice. Indeed, lOFC→BLA projections mediate reward value *learning* Malvaez et al., 2019. Third, although involving instrumental choices, the disconnection tasks included cues (e.g. objects, visual stimuli) associated with the actions and outcomes, such that OFC-BLA disconnection could have impacted the ability to use those cues to guide instrumental performance, similar to pathway-specific OFC circuit function Lichtenberg et al., 2021; Lichtenberg et al., 2017.

That the mOFC→BLA pathway is required for adjusting instrumental reward seeking based on the hunger-state-dependent incentive value of the predicted reward Malvaez et al., 2019 but not for sensitivity of instrumental choice to sensory-specific satiety devaluation Lichtenberg et al., 2021 is another seemingly contradictory set of results. This discrepancy may be explained by differences in the type of value learning. Incentive value is a long-term, consolidated, motivational state-dependent memory Dickinson and Balleine, 1994. Subjects learn the value of the reward in a particular state (e.g. hunger) and then 24 hr or more later are tested for their ability to use that information to guide their reward seeking. By contrast, the influence of sensory-specific satiety devaluation is typically tested immediately, with no opportunity for sleep or consolidation. The mOFC→BLA pathway is, therefore, important for using consolidated memories of the relationship between an internal physiological state and an expected outcome’s value to guide reward-pursuit decisions. This interpretation is consistent with mOFC→BLA function in the expression of outcome-specific PIT and sensitivity of Pavlovian conditional responses to devaluation, both of which require the use of consolidated external cue state memories to know which specific rewards are predicted. Thus, the mOFC→BLA pathway is important when previously learned states, whether internal or external, are needed to generate reward predictions. This implies that the mOFC→BLA pathway is recruited to support decision making with memory consolidation. This could be further tested by comparing mOFC→BLA pathway activity and necessity in instrumental choice following sensory-specific satiety devaluation with Balleine and Dickinson, 1998b and without the opportunity for memory consolidation.

## Orbitofrontal cortex – basolateral amygdala circuit function

The OFC-BLA circuit is critical for learning and memory processes that support decision making. There is a tendency to think BLA is primarily important for assigning general valence to predictive cues Pignatelli and Beyeler, 2019; Smith and Torregrossa, 2021; O’Neill et al., 2018; Correia and Goosens, 2016; Tye, 2018. It is. But, the above data reveal that the BLA, with support from OFC, helps to link information beyond valence, sensory-specific features of rewarding events to the external and internal states with which they are associated. And then, via its outputs to OFC to use that information to enable the predictions and inferences needed for flexible decision making. Thus, the BLA, via its connections with the OFC, is a critical contributor to decision making. The OFC has long been thought to support adaptive decision making. The data above reveal that many of these functions are supported via direct connections with BLA.

**Figure 1.**
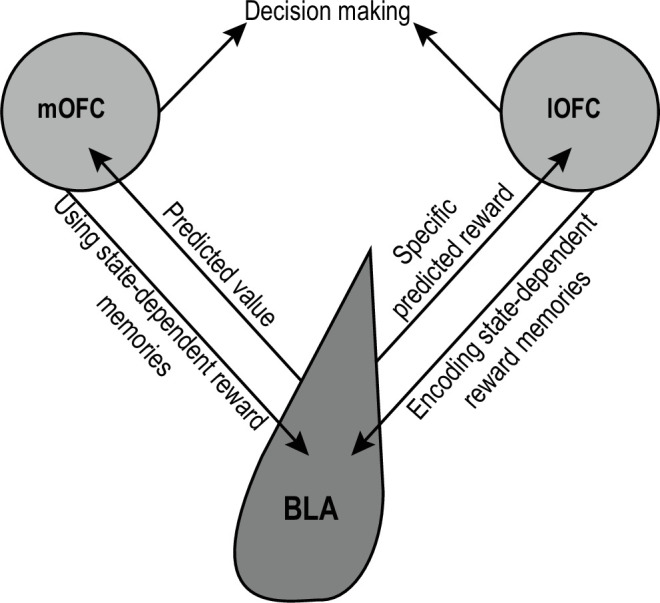
Schematic of OFC-BLA circuit function.

Each pathway in the OFC-BLA circuit makes a unique contribution to its overall function in forming state-dependent, outcome-specific reward memories and using this information to inform the predictions and inferences that guide reward-seeking decisions (Figure 1). When a rewarding event is experienced, activity in the lOFC→BLA pathway helps to link that specific reward to predictive states. For example, while eating the pizza you ordered via delivery, the lOFC→BLA pathway helps you link that specific pizza to the associated logos in the food-delivery app and to learn that meal is desirable when you are hungry. Later, activity in the mOFC→BLA pathway facilitates the ability to use these memories to guide decision making Sias et al., 2021; Malvaez et al., 2019; Lichtenberg et al., 2021. When you are hungry and see those logos in the future, the mOFC→BLA pathway helps you know pizza might be a good dinner option. Activity in BLA neurons projecting to the OFC enable state-dependent reward memories to guide decision making. BLA→lOFC projections contribute to using detailed representations of expected rewards to support decision making Sias et al., 2021; Lichtenberg et al., 2017. This pathway helps you to know what specific food is predicted by the restaurant logos (e.g. New York style pepperoni pizza). BLA→mOFC projections mediate the ability to adapt behavior based on the value of the predicted upcoming event Lichtenberg et al., 2021. This pathway helps you to know how desirable that pizza is, making you less likely to order it if you just had pizza for lunch. Together this circuit helps to form the associative memories we need to build an internal model of the world that we can later use to generate predictions about forthcoming events and inferences about how advantageous a certain course of action might be.

## Hypotheses and future directions

Recent work on the OFC-BLA circuit has opened many questions critical for understanding the function of this circuit and the neuronal substrates of appetitive associative memory and decision making more broadly.

### Neuronal encoding

Perhaps the most obvious question is the precise information content conveyed by each component of the OFC-BLA circuit and how it is used to shape neuronal encoding and representations in the receiving structure. Bulk activity recordings of each pathway will provide a useful entry point. Such investigations would benefit from multisite recordings to assess information flow across the circuit. A full understanding of OFC-BLA circuit function will, however, require cellular resolution investigation of each pathway’s activity during reward learning and decision making. These will, ideally, include pathway-specific manipulations to ask how each pathway contributes to the neuronal encoding downstream. These studies will have strong footing in the deep existing literature on the neuronal activity patterns of OFC and BLA Wassum and Izquierdo, 2015; Sharpe et al., 2019; Wikenheiser and Schoenbaum, 2016; Gardner and Schoenbaum, 2020; Wallis, 2011; O’Neill et al., 2018; Bissonette and Roesch, 2016; Salzman et al., 2007; Morrison and Salzman, 2010; Knudsen and Wallis, 2022; Enel et al., 2021; Sosa et al., 2021; Rich et al., 2018; Murray and Rudebeck, 2018; Averbeck and Costa, 2017; Sharpe and Schoenbaum, 2016. Several exciting hypotheses have emerged from these hub recordings and the pathway-specific functional investigations described above. Broadly, individual and/or ensembles of neurons in the OFC-BLA circuit are likely to be activated predictive states and to convey multifaceted information about predicted rewards, including their sensory-specific features and value, that is important for decision making. lOFC→BLA neurons might be activated by rewarding events during learning and encode information important for linking the sensory-specific and value features of those rewards to predictive states. mOFC→BLA neurons may carry information about reward-predictive states that relates to choices made in those states. BLA→lOFC projection neurons may show selective responses to unique reward-predictive cues and encode identifying features of the predicted reward and/or be required for such encoding in lOFC. BLA→mOFC projection neurons are also likely to be activated by reward-predictive cues and to either encode themselves or to facilitate encoding in mOFC of expected reward value.

### Mechanism

Of course, there are many levels at which mechanism can, and should, be explored. One possibility is that BLA cells that project to the lOFC and mOFC undergo synaptic, morphological, and/or molecular changes during learning to enable their function in state-dependent reward memory. Indeed, the ionotropic glutamate receptors known to regulate BLA synaptic plasticity Bauer et al., 2002; Müller et al., 2009 are required for encoding and using reward memories to guide decision making. An enticing hypothesis is that these neuroplastic changes are, at least in part, driven by lOFC→BLA input, and that mOFC→BLA inputs access activity in these neurons to mediate the ability to use predictive states to guide decision making. lOFC and mOFC axons are intermingled in the BLA Malvaez et al., 2019, but whether they make synaptic contact with the same cells or networks of cells is unknown. More broadly, information on direct and multisynaptic connections between each pathway is needed to better understand the extent and mechanisms of their interactions. The role of OFC and BLA interneurons will be important in this regard. It will also be important to explore the role of memory system consolidation in the neuroplastic changes that enable OFC-BLA circuit function. Although OFC-BLA projections are known to be excitatory, glutamatergic neurons Malvaez et al., 2019; Kita and Kitai, 1990; Hoover and Vertes, 2011; Heilbronner et al., 2016; Barreiros et al., 2021; Reppucci and Petrovich, 2016; Morecraft et al., 1992, little else is known about them. Whether the pathways between the OFC and BLA include molecularly-unique subpopulations and whether such potential populations are functionally distinct are ripe questions for future mechanistic investigation.

### Refining function

The tasks that have defined OFC-BLA circuit function all involved decisions in novel situations. For example, the PIT test is the first time subjects choose between the two actions and, moreover, those actions are unreinforced. Faced with these novel circumstances, subjects must use their knowledge of stimulus-outcome relationships to infer what to do. The incentive learning test requires subjects to pursue a reward for the first time while hungry. Following outcome-specific devaluation, the external environment is unchanged, but the internal state is new, the predicted reward is devalued. The OFC-BLA circuit is critical for the learning and memory processes that support decisions in these novel situations. Is this circuitry also involved in even more novel situations that require one to construct the value of a predicted reward on-the-fly using its attributes? Studies in humans suggest so. lOFC can represent an expected outcome’s constituent features Suzuki et al., 2017. The outcome’s value can be decoded from this information and is integrated to compute value in more medial cortical regions, including mOFC Suzuki et al., 2017. Is this circuitry involved in more well-practiced decision scenarios? Recent theories suggest perhaps not Gardner and Schoenbaum, 2020. OFC is needed for the learning that supports decision making, but not always for decision making itself Constantinople et al., 2019; Miller et al., 2020; Keiflin et al., 2013; Gardner et al., 2020. For example, neither lOFC nor mOFC are required for well-practiced, but still model-based, decisions Gardner et al., 2020. The extent to which novelty, inference, and on-the-fly decision making are critical features of OFC-BLA circuit function is a ripe question for future investigation.

Another critical question is whether the mOFC→BLA, BLA→lOFC, and BLA→mOFC pathways participate in memory retrieval v. the use of those memories to support decision making. That is, accessing memories of predicted rewards so they can be mentally represented v. using those representations to support the predictions and inferences that enable decisions. Given the BLA’s long-standing role in emotional memory Janak and Tye, 2015; Wassum and Izquierdo, 2015; LeDoux, 2000, it is a reasonable speculation that the BLA supports decision making, at least in part, via a memory retrieval process. One view is that memories are stored in the activity of ensembles of neurons Poo et al., 2016; Josselyn et al., 2015; Tonegawa et al., 2015. The BLA is one hub for this. Indeed, during fear conditioning the neuronal ensemble representing a cue becomes similar to that of the predicted aversive event. Thus, the BLA encodes the aversive association. These neurons are reactivated during memory retrieval Reijmers et al., 2007; Gore et al., 2015 and regulate the behavioral expression of that learning Han et al., 2009; Yiu et al., 2014. The information content of these BLA memory traces is not well known. Nonetheless, these findings suggest learning and memory retrieval processes might subserve BLA function and interactions with the OFC in decision making. However, the OFC is not required for well-practiced model-based decisions Gardner et al., 2020 that, presumably, require memory retrieval, but not on-the-fly inferences about option value. Thus, whereas the BLA may be important for retrieving reward memories, its projection to the OFC may be primarily important for using that information for the inferences that support decisions in novel situations. The BLA’s function in encoding and, likely, retrieving stimulus-outcome memories could serve other decision processes, including more practiced decisions, via alternate pathways including to the dorsal and ventral striatum and other cortical regions.

Many BLA-OFC pathway investigations capitalized on experimental control to parse reward learning from the use of this information to guide decisions. This enabled dissociation of function in learning (e.g. lOFC→BLA) v. using (mOFC→BLA) reward memories. But learning and decision making are often intertwined. For example, when cue- and action-reward contingencies are volatile. Reversal learning is one such dynamic scenario in which OFC, BLA, and lOFC→BLA projections have been implicated Groman et al., 2019a; Schoenbaum et al., 2002; Schoenbaum et al., 2003a; Burke et al., 2009; Izquierdo et al., 2013; Rudebeck and Murray, 2008; Churchwell et al., 2009; Chudasama et al., 2009; Butter et al., 1963; Boulougouris et al., 2007; Manning et al., 2021. More information is needed on the contribution of the OFC-BLA circuit to learning and decision making in dynamic and volatile situations.

Here I focused on state-outcome associative structures. These are important, but simple, components of the internal model of associative relationships that exist in the world. Environments often contain more complex and sequential structures. Particular actions are often needed to transition between states in these structures. For example, there are many intervening steps between seeing the pizza restaurant logo in a food-delivery app and actually eating the pizza. You select the pizza and place it in your cart, then check out, receive a notification that your order was placed, then picked up, then delivered, at which point you gather your meal, open the packaging, and then, finally, enjoy the pizza. Whether and how the OFC-BLA circuit participates in the encoding and use of complex sequential associations and the actions required to transition between states are important open questions. Evidence of hub function across species suggests the OFC-BLA circuit is likely involved. Human OFC activity can reflect multistage Pavlovian stimulus-stimulus contingencies Pauli et al., 2019 and encode a cognitive map of a complex state space Schuck et al., 2016. Non-human primate amygdala neurons can reflect plans in a multistage task Hernádi et al., 2015. In rodents, OFC dopamine tone correlates with model-based behavior in a multistage decision task Miller et al., 2020; Groman et al., 2019b and OFC inactivation disrupts model-based planning in such a task Miller et al., 2017. Even putatively single-step associative structures involving food reward (e.g. tone-pellet), such as those in which the OFC-BLA circuit was implicated above, actually include multiple state transitions. The tone signals the food, which can be more immediately signaled by tone offset and/or the subtle click of the pellet dispenser, food-port entry is required to transition from the state predicting the pellet to actually consuming it, the taste of the pellet itself predicts subsequent satiation. Thus, an important question for future investigation is the extent to which the OFC-BLA circuit contributes to encoding and using multistage associative models that are characteristic of model-based reinforcement learning and planning. In such investigations it will be important to evaluate whether OFC-BLA circuitry encodes each step in a multistage association and/or links initial predictive states to rewarding outcomes further away in the state space. Both navigational (e.g. maze) and multistage operant tasks will benefit these investigations Behrens et al., 2018. Of course, OFC-BLA circuit activity may not perfectly map onto existing model-based reinforcement learning structures, but such structures will provide a crucial theoretical framework.

### Generalizing function

Another important question is whether OFC-BLA circuit function in encoding state-dependent, outcome-specific memories and using such memories to guide decision making applies to the aversive domain. It does seem plausible, if not likely. Like the BLA, both lOFC and mOFC contribute to aversive behavior Orsini et al., 2015; Plassmann et al., 2010; Zimmermann et al., 2018; Ma et al., 2020; Verharen et al., 2019; Turner et al., 2021; Jean-Richard-Dit-Bressel and McNally, 2016; Ishikawa et al., 2020; Shih and Chang, 2021; Metereau and Dreher, 2015; O’Doherty et al., 2001; Fullana et al., 2016. lOFC activity influences sensitivity to punishment. In some cases, it is important for guiding choices away from punishment Jean-Richard-Dit-Bressel and McNally, 2016. In others, it is important for pursuing reward despite risk of punishment Orsini et al., 2015; Ishikawa et al., 2020. mOFC is critical for sensitivity to punishment Ma et al., 2020; Verharen et al., 2019, especially when it is infrequent requiring subjects to rely on their memory of the aversive outcome Ma et al., 2020. Both lOFC and mOFC are also needed to use contexts to know when aversive events are and are not expected Shih and Chang, 2021. Thus, OFC is involved in making choices based on both potential appetitive and aversive outcomes. Whether the OFC-BLA circuit mediates state-dependent, outcome-specific aversive memories and their influence over decision making is, thus, a ripe question. To answer this question, it will be important to assess outcome-specific aversive memories. This has been procedurally difficult. Classic outcome revaluation tasks from Rescorla, 1973; Rescorla, 1974 and aversive PIT Lewis et al., 2013; Campese, 2021 will be a good start. These investigations will also benefit from consideration of the procedural differences between aversive and appetitive learning. For example, aversive learning typically involves far fewer training trials and days than appetitive learning. Aversive shocks can be immediately delivered, whereas appetitive outcomes typically have to be collected from a delivery port. There may also be inherent differences in the nature of the outcomes. Foods produce a taste and later satiation. Aversive events produce an immediate aversive experience that can have longer-lasting emotional consequences. Such differences are likely to contribute to the neuronal circuitry involved.

The BLA and OFC have also been implicated in learning about different types of rewarding events Wassum and Izquierdo, 2015; Rosenberger et al., 2019; Walum and Young, 2018; Song et al., 2021. So, it will be also interesting to explore the extent to which the OFC-BLA circuit supports the encoding and use state-dependent, outcome-specific memories of non-food rewards, including social interactions and addictive substances. These investigations will also benefit from new methods to access memory content, state dependency, and inference.

Of course, it will also be important to uncover how the OFC-BLA circuit works with broader cortical-thalamic-basal ganglia systems to support learning and decision making. For example, it will be interesting to know whether the BLA supports other prefrontal cortex regions in their contributions to decision making in a manner similar to its support of OFC. Likewise, it will be important to know what other subcortical regions support the OFC in learning and decision making. BLA and OFC interactions at the level of the striatum, a major interface for action execution, is also an important avenue for investigation. In understanding the broader circuit, it will help to know whether the architecture exposed here relates to other bidirectional corticolimbic circuits. For example, are there other corticolimbic systems with separate learning v. retrieval input channels or top-down learning signals that drive bottom up retrieval?

### Implications for learning and decision models

These neurobiological investigations have implications for our understanding of the psychological processes that control learning and decision making.

A reward’s identity can be neurobiologically dissociable from its value. When the BLA→mOFC pathway is inactivated subjects can use cues to represent the identity of the predicted reward (needed to express outcome-specific PIT) but cannot represent its value (needed for sensitivity of the conditional response to devaluation). Thus, reward identity and value are likely separate nodes in the associative structure that animals use to allow cues to generate predictions for adaptive behavior and choice.

External and internal states may share some associative coding structure. The states that access information about reward identity and value can be both external (i.e. environmental cues) and internal (e.g. physiological, homeostatic signals). The encoding and use of both forms of memory have partially overlapping neuronal substrates: lOFC→BLA and mOFC→BLA pathways. There are neurobiological similarities in how we learn that a logo predicts a specific food and that a particular food will be tasty when we are hungry. Thus, there may be associative coding structures that support both state types. External and internal state information could converge in the BLA-OFC circuit or could be coded in different streams, perhaps defined by different cell types, within the circuit. Regardless, external and internal states are poised to interact in the OFC-BLA circuit. Indeed, the BLA receives and integrates information about external cues and internal homeostatic states Livneh et al., 2017.

### Implications for maladaptive learning and decision making

Deficits in the ability to learn and/or use information about expected rewarding outcomes can lead to ill-informed decisions and this is characteristic of the cognitive symptoms that can underlie several psychiatric illnesses, including substance use disorder Hogarth et al., 2013; Dayan, 2009; Voon et al., 2015; Schoenbaum et al., 2016; Everitt and Robbins, 2016; Volkow et al., 2013, depression Seymour and Dolan, 2008; Heller et al., 2018; Chen et al., 2015; Huys et al., 2015, anxiety Alvares et al., 2014, and schizophrenia Morris et al., 2015; Culbreth et al., 2016. These conditions have also been associated with altered activity in BLA and OFC as well as OFC-BLA connectivity Ressler and Mayberg, 2007; Price and Drevets, 2010; Sladky et al., 2015; Liu et al., 2014; Passamonti et al., 2012; Goldstein and Volkow, 2011; Tanabe et al., 2009; Linke et al., 2012; Hahn et al., 2011; Xie et al., 2021. Thus, OFC-BLA circuit dysfunction might underlie some of the learning and decision-making symptoms of substance use disorder and other mental illnesses. The above data exposed vulnerabilities in the circuit whereby disrupted activity might cause maladaptive decision making. For example, one may be able to know which rewards are available but unable to understand their current value (e.g., BLA→mOFC dysfunction). This could lead to continued drug pursuit despite negative consequences or, conversely, lack of motivation for actions that earn valuable outcomes, despite knowledge of those outcomes (e.g. consuming healthy food or going to work). Or one might have learned about a predicted reward but be unable to use that memory to inform choices in the moment (mOFC→BLA dysfunction). For example, one may have learned about the negative consequences of a drug, or positive effects of eating healthy foods, but be unable to use that information when presented with drug or food cues, leading to poor decisions. Further understanding of the function of the OFC-BLA circuit in both adaptive and maladaptive decision making is likely to aid our understanding and treatment of substance use disorder and other mental illnesses.

### Conclusion

The OFC-BLA circuit helps us to encode detailed, outcome-specific memories of rewarding events and to access those memories under the right circumstances to enable the predictions and inferences that support adaptive decision making. There is much to be learned about the precise function of each pathway, information flow through the circuit, and the extent to which the circuit function generalizes to other types of outcomes. More mechanistic insight is clearly needed. Yet, the recent investigations make clear that the OFC-BLA circuit is a critical contributor to learning and memory processes that underlie the considerations we use to make daily decisions and that are disrupted in myriad psychiatric diseases.
